# Chilaiditi's Syndrome Treatment Using Versius Robotic Surgical System: A Case Report

**DOI:** 10.1155/cris/9965465

**Published:** 2025-06-12

**Authors:** Daniele Sandonà, Diego Caroli, Giacomo Sarzo, Enzo Mammano

**Affiliations:** ^1^Department of Surgical Oncological and Gastroenterological Sciences – DiSCOG, University of Padua, Padua, Italy; ^2^Department of Gastroenterology AOPD, University of Padua, Padua, Italy; ^3^Department of Surgery, Hospital Sant' Antonio AOPD, University of Padua, Padua, Italy

## Abstract

**Introduction:** Chilaiditi's sign consists of the interposition of a segment of the intestine between the right diaphragm and the liver; when this anomaly causes gastrointestinal symptoms, it is referred to as Chilaiditi's syndrome. If conservative treatment fails, surgical intervention is often necessary, especially in severe or complicated cases.

**Case Presentation:** An 82-year-old woman with a 2-year history of right-sided abdominal pain, constipation, malaise, and weight loss was diagnosed with Chilaiditi's syndrome following an extensive workout to exclude other pathologies. Following the failure of medical therapy, she underwent elective robotic surgery for hepatic flexure mobilization and right colopexy. The procedure was performed using the Versius robotic system (Cambridge Medical Robotics, CMR), resulting in successful repositioning of the colon and resolution of symptoms.

**Discussion:** Chilaiditi's syndrome is often underdiagnosed and can be effectively treated with surgical intervention in cases unresponsive to medical therapy. The Versius robotic system offers a highly effective, minimally invasive solution, reducing surgical trauma, and promoting faster recovery. This case highlights the benefits of robotic-assisted surgery in managing complex gastrointestinal conditions like Chilaiditi's syndrome.

**Conclusion:** Robotic surgery, particularly with the Versius robotic system, offers significant technical advantages in such complex cases due to its precision and minimally invasive nature, with improved clinical outcomes, and enhanced recovery times for patients requiring surgical intervention.

## 1. Introduction

Chilaiditi's sign is a radiological finding characterized by the interposition of the colon between the liver and the right hemidiaphragm. This rare anomaly is typically seen incidentally on chest or abdominal radiographs, with a reported incidence of 0.025%–0.28%. Chilaiditi's syndrome is diagnosed when this anatomical variation results in gastrointestinal symptoms [1]. This condition is a potentially treatable cause of abdominal pain and other gastroenterological symptoms. The first-line treatment for patients with this syndrome consists of medical therapy, while surgical intervention is the most appropriate treatment option for those who do not respond to medical treatment [2]. Minimally invasive surgery is considered the best choice for these patients [3, 4]. This report is the first to describe the use of the Versius robotic system for the management of this rare syndrome. This work has been reported in-line with the SCARE criteria [5].

## 2. Case Presentation

**Figure 1:**
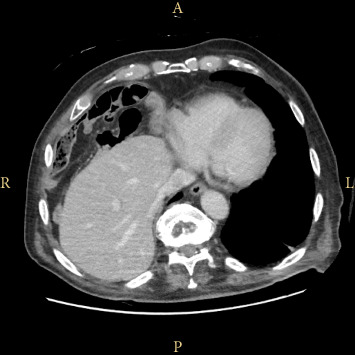
Axial CT scan showing Chilaiditi's sign; A (anterior), P (posterior), L (left), R (right).

**Figure 2:**
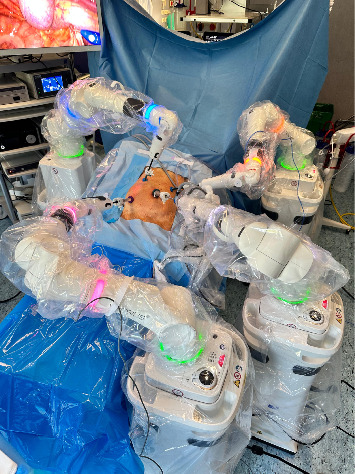
Docking of four Versius robotic arms at the beginning of the procedure: blue arm holds a cadiere, pink arm holds a bipolar, white arm holds the camera, and the orange arm holds a monopolar scissor.

**Figure 3:**
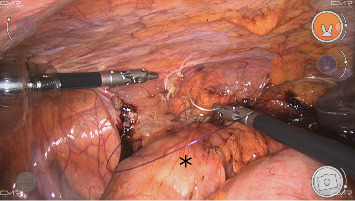
An intraoperative image shows the robotic colopexy of the right colon (black asterisk) with a running barba suture.

An 82-year-old nonsmoker woman with a normal weight (BMI: 23) presented to the outpatient clinic with a 2-year history of right upper and lower quadrant abdominal pain, associated with constipation, malaise, and a 6 kg weight loss over the previous year (current weight: 55 kg). The patient's medical history was significant for atrial fibrillation, arterial hypertension, appendectomy, and laparoscopic ovarian cyst removal. She has no allergies, and her medical therapy consisted of oral antihypertensive and anticoagulant drugs. Laboratory tests showed no abnormalities. A colonoscopy revealed only mild sigmoid diverticulosis and a small tubular adenoma in the rectum with low-grade dysplasia. Abdominal computed tomography (CT) scan showed classic features of Chilaiditi's sign, with a loop of colon positioned between the liver and the right hemidiaphragm (Figure 1). All other possible etiologies were ruled out. She was initially treated with a high-fiber diet, laxatives, and antispasmodics. After medical therapy failure, the patient was scheduled for elective robotic mobilization of the hepatic flexure and right colopexy. During the procedure, the patient was placed in the supine position, with 10° anti-Trendelenburg tilt and 5° left lateral rotation. Access to the abdominal cavity was achieved using the Hasson technique, with three other trocars positioned under direct vision: One 12 mm trocar in the right iliac fossa, 5 mm in the left flank, and 5 mm in the right hypochondrium. Four Versius robotic arms were then docked (Figure 2). The hepatic flexure, which was located above the hepatic margin, was isolated and detached until the right hemidiaphragm was exposed. The colon was then repositioned below the hepatic margin. A running barbed suture was used to fix the right colon and cecum to the right parietocolic gutter, restoring them to the anatomically correct position (Figure 3). No postoperative drain was placed. The procedure lasted 101 min with a console time of 64 min. The postoperative course was uneventful, and the patient was discharged 2 days after surgery. Quality of life improvement was assessed by administering the Gastrointestinal Quality of Life Index (GIQLI) questionnaire, which improved from 74 preoperatively to 124 1 month postoperatively. At 3 month follow-up, there were no complications, the patient's satisfaction was high, and she regained 1 kg.

## 3. Discussion

Chilaiditi's syndrome refers to the clinical condition in which Chilaiditi's sign is accompanied by clinical symptoms. Considering current evidence, smoking status doesn't have an impact on these patients. Intestinal, hepatic, and/or diaphragmatic etiologies contribute to the pathogenesis of both Chilaiditi's sign and the syndrome of the same name [1]. Our patient reported symptoms that significantly impacted her quality of life. In patients with Chilaiditi's syndrome, the most common symptoms are gastrointestinal (e.g., abdominal pain, nausea, vomiting, and constipation), followed by respiratory distress and less frequently, angina-like chest pain. Rarely, these symptoms may coexist. The severity of the symptoms can vary, with the most serious cases leading to acute abdomen. Differential diagnosis is often challenging, requiring a thorough diagnostic workup, as in our case, where definitive surgical treatment occurred 18 months after initial evaluation. Surgery is indicated if the patient does not respond to initial conservative treatment, if obstruction persists, or if there are signs of intestinal ischemia [2]. Among the procedures described in the literature, we selected hepatic flexure mobilization and colopexy. We opted for a minimally invasive robotic technique using the Versius robotic system, which has proven to be safe and effective in minimally invasive surgery. The major advantages that led us to select this system include its ergonomic platform, which reduces stress and fatigue for the surgeon, the V-wrist technology that allows 360-degree movement of the wrist, and the ability to freely position the ports thanks to the individual robotic arms, which facilitate replicating laparoscopic steps [6]. The Versius robotic surgical system was particularly beneficial due to the need for long sutures required for colopexy and the presence of separate robotic arms. These arms enabled a surgeon with considerable laparoscopic experience to successfully perform a complex robotic procedure, even though he was at the early stages of his robotic experience. Furthermore, the results were evaluated using the GIQLI score, a well-established tool in the literature [7]. This demonstrated a clear improvement 1 month postsurgery. The patient had no complications at the 3 month follow-up and continued to be highly satisfied with the procedure.

## 4. Conclusion

Chilaiditi syndrome is a rare clinical condition, and due to its low incidence, observational studies are currently lacking. This case report highlights the use of a novel robotic device for the treatment of this syndrome. The Versius robotic system provides significant advantages for the surgeon, including enhanced ergonomics and versatility, while also ensuring excellent postoperative outcomes for the patient.

## Data Availability

The data that support the findings of this study are available upon request from the corresponding author. The data are not publicly available due to privacy or ethical restrictions.
